# Chemical Profiling and Molecular Docking Study of *Agathophora alopecuroides*

**DOI:** 10.3390/life12111852

**Published:** 2022-11-11

**Authors:** Elham Amin, Mohamed Sadek Abdel-Bakky, Hamdoon A. Mohammed, Marwa H. A. Hassan

**Affiliations:** 1 Department of Medicinal Chemistry and Pharmacognosy, College of Pharmacy, Qassim University, Buraydah 51452, Saudi Arabia; 2 Department of Pharmacognosy, Faculty of Pharmacy, Beni-Suef University, Beni-Suef 62514, Egypt; 3 Department of Pharmacology and Toxicology, College of Pharmacy, Qassim University, Buraydah 51452, Saudi Arabia; 4 Department of Pharmacology and Toxicology, Faculty of Pharmacy, Al-Azhar University, Cairo 11751, Egypt; 5 Department of Pharmacognosy and Medicinal Plants, Faculty of Pharmacy, Al-Azhar University, Cairo 11751, Egypt

**Keywords:** *Agathophora alopecuroides*, antidiabetic, LC-HRMS/MS, molecular docking

## Abstract

Natural products continue to provide inspiring chemical moieties that represent a key stone in the drug discovery process. As per our previous research, the halophyte *Agathophora alopecuroides* was noted as a potential antidiabetic plant. However, the chemical profiling and highlighting the metabolite(s) responsible for the observed antidiabetic activity still need to be investigated. Accordingly, the present study presents the chemical profiling of this species using the LC-HRMS/MS technique followed by a study of the ligand–protein interaction using the molecular docking method. LC-HRMS/MS results detected twenty-seven compounds in *A. alopecuroides* extract (AAE) belonging to variable chemical classes. Among the detected compounds, alkaloids, flavonoids, lignans, and iridoids were the most prevailing. In order to highlight the bioactive compounds in AAE, the molecular docking technique was adopted. Results suggested that the two alkaloids (Eburnamonine and Isochondrodendrine) as well as the four flavonoids (Narirutin, Pelargonidin 3-O-rutinoside, Sophora isoflavanone A, and Dracorubin) were responsible for the observed antidiabetic activity. It is worth mentioning that this is the first report for the metabolomic profiling of *A. alopecuroides* as well as the antidiabetic potential of Isochondrodendrine, Sophora isoflavanone A, and Dracorubin that could be a promising target for an antidiabetic drug.

## 1. Introduction

Diabetes mellitus is a chronic metabolic disease recognized by an increase in blood glucose levels which develops from a deficiency in insulin secretion, action, or both of them [1]. Type 2 diabetes mellitus (T2DM) is the most common health problem and accounts for about 90% of diabetes cases with 4.9 million mortalities throughout the world [2]. Inhibiting the digestion of dietary carbohydrates is one of the effective procedures for the management of postprandial hyperglycemia in T2DM. One of the essential digestive enzymes is pancreatic α-amylase which converts dietary carbohydrates such as starch into smaller oligosaccharides mixture that are further broken down into glucose by α-glucosidase, another important metabolic enzyme. Upon the absorption of glucose, it enters the bloodstream and causes postprandial elevation in blood glucose levels. Therefore, blocking the enzymes α-amylase and α-glucosidase can inhibit the digestion of carbohydrates, postpone glucose uptake, and subsequently lower blood sugar levels [3]. Recently, medicinal plants proved their great therapeutic potential and negligible side effects in the treatment of T2DM. For instance, several medicinal herbs have been reported to exhibit strong glucosidase and amylase inhibitory properties [4,5,6].

Halophytes are salt-tolerating plants noted for their ability to produce variable secondary metabolites, such as alkaloids, glycosides, and terpenes. Hence, they could be considered as promising sources for bioactive metabolites that could be used for the treatment of various diseases such as diabetes [7,8,9]. *A. alopecuroides* is a halophytic species prevalent in the deserts of Saudi Arabia and was reported to exhibit a strong in vitro and in vivo antidiabetic activity [7]; nevertheless, its chemical profile remained to be investigated [10].

Dereplication, defined as the rapid identification of known compounds from natural product extracts, represents an important step in drug discovery programs. This approach combines the benefits of different analytical techniques, modern spectroscopic methods, as well database searches for the prompt characterization of an active compound during the drug discovery process. Recently, advances in technology have provided what is called tandem analytical techniques, such as LC-MS, LC-MS/MS, LC-NMR, HPLC-PDA, and LC-NMR/MS [11]. Tandem mass spectrometry (MS/MS) is a powerful technique for the characterization of target phytoconstituents in complex plant extracts. The high sensitivity, selectivity, and fast screening abilities of the LC-MS/MS technique, compared to other dereplication techniques, rationalized the privilege of this technique for the online identification of secondary metabolites in plant extracts [11].

Furthermore, advances in computational biology have had a great impact on reducing the time, cost, and effort spent while screening the biological activity of natural products. Molecular docking is now widely adopted for predicting the binding mode and binding affinity of a drug-like molecule into the active site of the receptor. A huge number of natural and synthetic compounds could be virtually screened for activity against a wide array of targets, thus reducing the time and effort and giving a rapid expectation for the most promising candidates [12].

Based on this concept and in continuation of our previous research on the halophytes with promising antidiabetic potential [7], which recorded the strong in vitro and in vivo antidiabetic potential of AAE, the current study investigated the metabolic content of AAE using LC-MS/MS followed by the screening of the annonated compounds for the enzyme inhibitory potential against the two carbohydrate-metabolizing enzymes—α-amylase and α-glucosidase—using the molecular docking technique.

## 2. Materials and Methods

### 2.1. Plant Materials

*Agathophora alopecuroides* var. papillosa was collected from the Qassim region in the northcentral Saudi Arabia during October 2021. The taxonomic identity of the plant was confirmed by Ibrahim Aldakhil, botanical expert, Qassim Area, and avoucher sample number QPP-101 was kept at the College of Pharmacy, Qassim University (Buraydah, Qassim, Saudi Arabia). The aerial parts of the plant were carefully cleaned then dried in the shade for two weeks. The dried powdered plant material (500 g) was extracted by maceration in 80% methanol (3 times × 1000 mL) at room temperature with frequent shaking. The methanolic extract was then concentrated by a vacuum rotary evaporator. The dried extract was then kept in an amber-colored vial at 4 °C till further use.

### 2.2. Metabolites Profiling of the Methanolic Extract of A. alopecuroides

AAE was reconstituted in HPLC grade methanol, filtered through a 0.22 µm PTFE membrane, and then separation was performed adopting Thermo Scientific C_18_ column (AcclaimTM Polar Advantage II, 3 × 150 mm, 3 µm particle size) on an UltiMate 3000 UHPLC system (Dionex). Gradient elution was performed at a flow rate of 0.4 mL/min and a column temperature of 40 °C, using H_2_O + 0.1% Formic Acid (A) and 100% Acetonitrile (B) with a 22 min total run time. The injection volume of the sample was 3 µL. The gradient started at 5% B (0–3 min), 80% B (3–10 min), 80% B (10–15 min), and 5% B (15–22 min). High-resolution mass spectrometry was carried out using a MicroTOF QIII (Bruker Daltonic, Bremen, Germany) using an ESI positive ionization and adjusting the following settings: capillary voltage: 4500 V; nebulizer pressure: 2.0 bar; drying gas: 8 L/min at 300 °C. The mass range was 50–1000 *m*/*z*. The accurate mass data of the molecular ions, provided by the TOF analyzer, were processed by Compass Data Analysis software (Bruker Daltonik GmbH).

### 2.3. Molecular Docking Study

AutoDock Vina software was used in all molecular docking experiments [13]. All dereplicated compounds were docked against the active sites of both human α-amylase and human α-glucosidase crystal structure (PDB codes: 4W93 and 3L4W, respectively) [14,15]. The binding site was determined according to the enzyme’s co-crystallized ligands (Montbretin A and Miglitol, respectively). The co-ordinates of the grid boxes were (x = −9.682; y = 4.274; z = −23.145 and x = 45.424; y = 92.375; z = 34.811). The size of the grid box was set to 20 Å. Exhaustiveness was set to 24. Ten poses were generated for each docking experiment. Docking poses were analyzed and visualized using Pymol software [13]. The full method is provided in the Supplementary Materials.

## 3. Results

### 3.1. In Vitro Testing of the Antidibetic Activity of A. alopecuroides (AAE)

This research represents an extension of our previous work on halophytic plants with potential antidiabetic activity. In the previous publication, in vitro testing revealed the strong inhibitory activity of the hydroalcoholic extract (AAE) against α-glucosidase and α-amylase with IC_50_ values 117.9 and 90.9 µg/mL, respectively, compared to 191.4 and 53.3 µg/mL of the standard drug Acarbose [7].

### 3.2. Metabolomic Profiling of the Methanolic Extract of A. alopecuroides (AAE)

**Figure 1:**
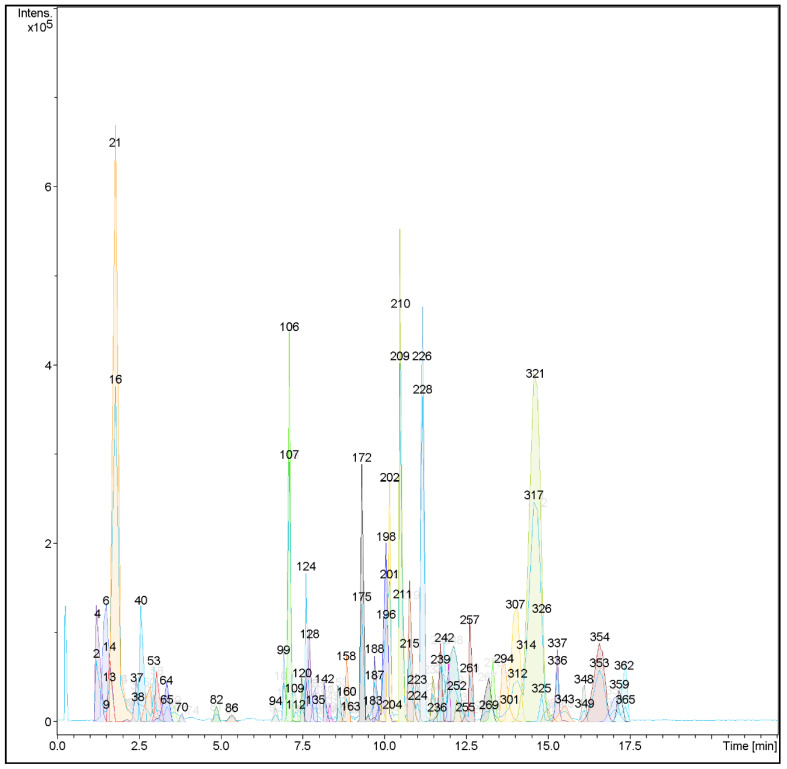
LC-HRMS chromatogram of the dereplicated metabolites of *A. alopecuroides* methanolic extract (positive mode).

**Figure 2:**
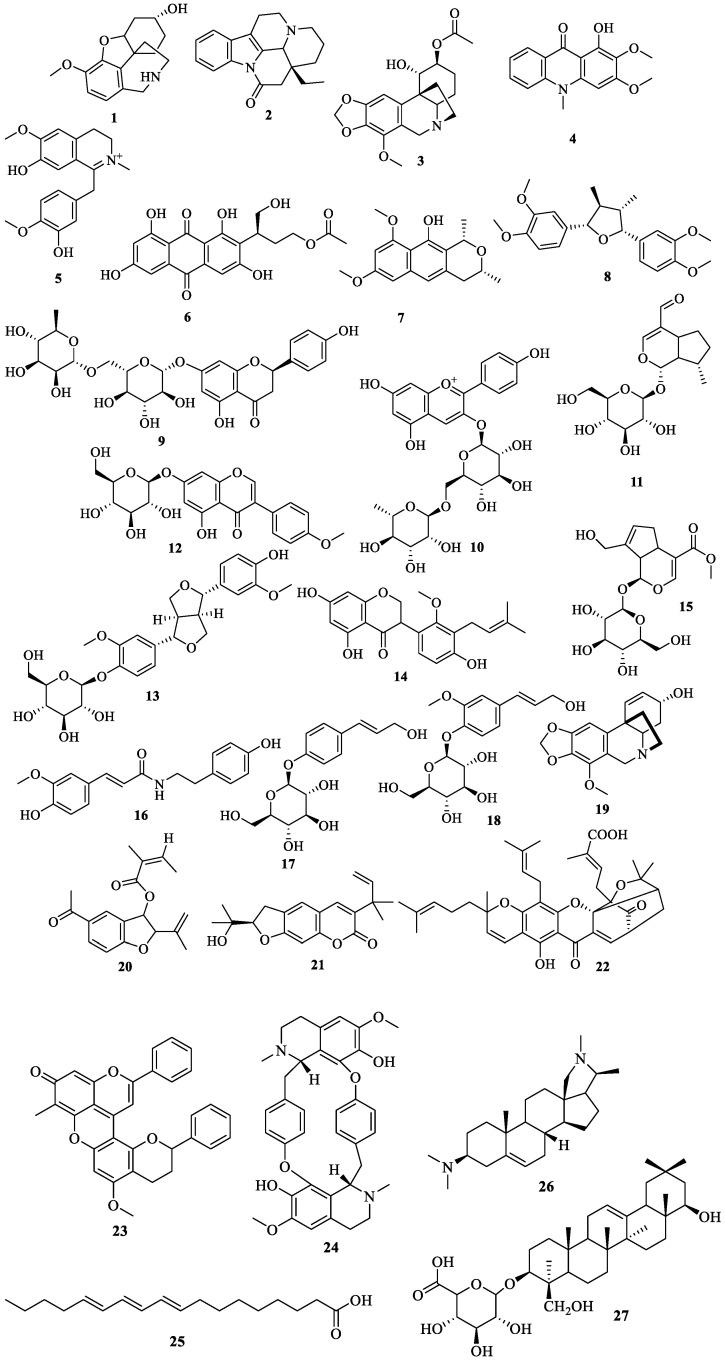
Structures of the dereplicated compounds from *A. alopecuroides* methanolic extract by LC-HRMS/MS.

The metabolic profiling using LC-HRMS/MS of AAE led to the annonation of 27 metabolites with variable chemical structures. The annonated metabolites could be classified according to their chemical classes into: nine alkaloids, five flavonoids, four lignans, two iridoid glycosides, two acids, one anthraquinone, one benzisochroman, one furanochomarine, one triterpenoid saponin, and one benzofuran (Table 1, Figure 1 and Figure 2). Using the metabolomic data, the nine alkaloids were annotated from the mass ion peaks at *m*/*z* 275.152, 294.173, 361.153, 285.290, 328.155, 313.131, 301.131, 594.273, and 356.319, which were in agreement with the molecular formulas C_16_H_21_NO_3_, C_19_H_22_N_2_O, C_19_H_23_NO_6_, C_16_H_15_NO_4_, C_19_H_22_NO_4_, C_18_H_19_NO_4_, C_17_H_19_NO_4_, C_36_H_38_N_2_O_6_, and C_24_H_40_N_2,_ respectively. These alkaloids were dereplicated as Epinorlycoramine (**1**), Eburnamonine (**2**), 3-Acetylnerbowdine (**3**), Arborinine (**4**), 1,2-Dehydroreticuline (**5**), N-Feruloyltyramine (**16**), Powelline (**19**), Isochondrodendrine (**24**), and Conessine (**26**), respectively. While the five flavonoids were dereplicated from the mass ion peaks at *m*/*z* 580.179, 579.171, 446.121, 370.142, and 488.162 that matched with the molecular formulas C_27_H_32_O_14_, C_27_H_31_O_14_, C_22_H_22_O_10_, C_21_H_22_O_6_, and C_32_H_24_O_5_, respectively. These metabolites were characterized as Narirutin (**9**), Pelargonidin 3-O-rutinoside (**10**), Biochanin A-β-D-glucoside (**12**), Sophora isoflavanone A (**14**), and Dracorubin (**23**), respectively. The four lignans were dereplicated from the molecular ion peaks at *m*/*z* 372.194, 520.194, 312.121, and 342.131 corresponding to the suggested formulas C_22_H_28_O_5_, C_26_H_32_O_11_, C_15_H_20_O_7_, and C_16_H_22_O_8,_ respectively, as Veraguensin (**8**), Pinoresinol glucoside (**12**), 4-Hydroxycinnamyl alcohol 4-D-glucoside (**17**), and Coniferin (**18**), respectively. The mass ion peaks at *m*/*z* 344.147 and 388.137 were in agreement with the molecular formulas C_16_H_24_O_8_ and C_17_H_24_O_10_ that were characterized as the two iridoid glycosides Boschnaloside (**11**) and Geniposide (**15**). Additionally, seven mass ion peaks at *m*/*z* 402.095, 288.136, 300.136, 314.152, 628.304, 278.225, and 634.408, compatible with the molecular formulae C_20_H_18_O_9_, C_17_H_20_O_4_, C_18_H_20_O_4_, C_19_H_22_O_4_, C_38_H_44_O_8_, C_18_H_30_O_2_, and C_36_H_58_O_9_, respectively, were dereplicated as Versiconol acetate (**6**), Karwinaphthol B (**7**), Toxyl angelate (**20**), Heliettin (**21**), Gambogic acid (**22**) Punicic acid (**25**), and Soyasapogenol B 3-*O*-d-glucuronide (**27**), respectively.

### 3.3. Molecular Docking Analysis

**Figure 3:**
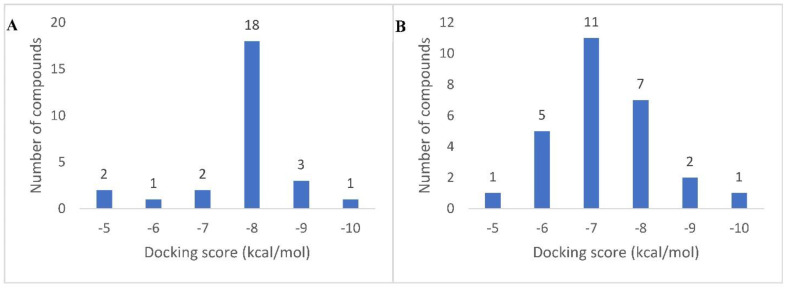
Docking score distribution of the dereplicated metabolites in AAE against both human α-amylase (**A**) and α-glucosidase (**B**) (PDB: 4W93 and 3L4W, respectively).

All the dereplicated compounds were subjected to molecular docking against both human α-amylase and human α-glucosidase enzymes (PDB codes: 4W93 and 3L4W, respectively). As shown in (Table 2 and Figure 3) and (Supplementary Materials, Figures S1 and S2), all dereplicated compounds received scores ranging from ~−4.5 to −9.1 kcal/mol against both enzymes. Most of the compounds (21 compounds in the case of α-amylase, and 23 compounds in the case of α-glucosidase) received scores ranging between −5 and −8 kcal/mol. The compounds that achieved the best score (<−8 kcal/mol) against α-amylase enzyme were Eburnamonine (**2**), Narirutin (**9**), Pelargonidin 3-*O*-rutinoside (**10**), and Isochondrodendrine (**24**). Considering α-glucosidase enzyme inhibition, the best scoring compounds (<−8 kcal/mol) were Pelargonidin 3-*O*-rutinoside (**10**), Sophora isoflavanone A (**14**), and Dracorubin (**23**). The two co-crystallized compounds—Montbretin A with amylase and Miglitol with glucosidase—achieved binding scores of −8.1 and −8.0 kcal/mol, respectively, and hence −8.0 kcal/mol was chosen as a cut-off value to select the best scoring compounds with each enzyme.

## 4. Discussion

This study is a continuation of our previous research dealing with the potential antidiabetic agents from halophytes. Previously, AAE was acknowledged for potent antidiabetic activity through both in vitro and in vivo investigations. The current study adds more information about the metabolic profiling of AAE and highlights the most promising metabolites expected to be responsible for the recorded antidiabetic activity.

**Metabolomic profiling of the methanolic extract of *A. alopecuroides***

For tentative identification of the components of AAE, we adopted a dereplication strategy using the hyphenated technique: liquid chromatography coupled with tandem mass spectrometry (LC-HRMS/MS) (Figure 1 and Figure 2). The dereplication process led to the recognition of 27 compounds for the first time from AAE. The detected metabolites belong to different chemical classes: alkaloids, flavonoids, lignans, iridoid glycosides, anthraquinones, benzisochromans, furanochomarine, triterpenoid saponins, acids, and benzofurans (Table 1, Figure 2). Amid the detected metabolites, alkaloids represent the most prevailing chemical class. These alkaloids belong to different types, and among them the most abundant is the isoquinoline-type alkaloids (Figure 1). Epinorlycoramine (**1**) is a galanthamine-type alkaloid previously isolated from *Narcissus leonensis* whole plant [16], while Eburnamonine (**2**), an eburnan-type alkaloid, was isolated from *Kopsia larutensis* leaves [17]. From the crinine-type alkaloids, two alkaloids were detected: 3-Acetylnerbowdine (**3**), which was reported in *Nerine bowdenii* bulbs using GC-MS analysis [18,19], and Powelline (**19**), reported in *Crinum latifolium* leaves using GC-MS analysis [40]. Arborinine (**4**), a nitrogen-containing drug similar to anthracene classified as acridone alkaloid, was previously isolated from the ethyl acetate extract of the *Glycosmis parva* plant [20]. The quaternary isoquinoline alkaloid 1,2-Dehydroreticuline (**5**), previously isolated from *Xylopia parviflora* root and bark [21,22], was also identified. N-Feruloyltyramine (**16**) is a phenolic amide alkaloid previously detected in *Lycium barbarum* fruits and *A. alopecuroides* [10,35]. Isochondrodendrine (**24**) is bis benzyli-soquinolinic alkaloids previously reported in the *Cissampelos pareira* plant [46,51]. Finally, Conessine (**26**), a pentacyclic steroidal alkaloid, has been isolated from *Holarrhena floribunda* G. Don. [48]. 

Another biologically important and widely prevailing chemical class is the flavonoids that were represented by five compounds from different flavonoid subclasses. Narirutin (**9**) is a flavanone common in the citrus family and reported for potent antidiabetic activity using in vitro and docking studies [27]. Pelargonidin 3-*O*-rutinoside (**10**), an anthocyanin with potent antidiabetic activity depicted through the inhibition of α-glucosidase and α-amylase enzymes, was isolated from strawberries [28]. Biochanin A-β-d-glucoside (**12**) is an isoflavone previously isolated from *Trifolium pratense* L. [31], while Sophora isoflavanone A (**14**) is a pterocarpan previously identified from *Sophora tomentosa* [33]. Finally, Dracorubin (**23**) was recognized as the major red coloring matter in the tree *Dracaena draco* resin [45].

LC-HRMS/MS results also characterized four lignans. Veraguensin (**8**) is a lignan compound previously identified in *N. turbacensis* (Kunth) Nees leaves and root bark [25,26]. Pinoresinol glucoside (**12**) was isolated from *Prunus domestica* [32] and was stated to exhibit potent antioxidant activity and powerful in vitro antihyperglycemic and hepatoprotective effects. 4-Hydroxycinnamyl alcohol 4-d-glucoside “4-*O*-β-d-glucopyranosyl-*p*-coumaric acid” (**17**) is a phenolic acid derivative that was isolated and identified in the flaxseed phenolic rich fraction [36,37]. Coniferin (**18**), is a phenolic glycoside previously isolated from the bark of *Paulownia tomentosa* [38,39].

Furthermore, the two iridoid glycosides Boschnaloside (**11**), previously reported from *Euphrasia pectinata* aerial parts [29,30], and Geniposide (**15**), isolated from *Gardenia jasminoides* Ellis fruit [34], were detected in AAE. 

An additional seven compounds from variable secondary metabolites classes were also detected in AAE and dereplicated as: the anthraquinone compound Versiconol acetate (**6**), previously recognized in the cultures of *Aspergillus parasiticus* after using the insecticide dichlorvos [52,53]; the dimethyl benzisochroman compound Karwinaphthol B (**7**), isolated from *Karwinskia humboldtiana* roots [24]; the natural benzofuran Toxyl angelate (**20**), previously isolated from *Isocoma wrightii* plant [41]; the furanocoumarine compound Heliettin (**21**), previously isolated from the stem bark of *Helietta longifolia* Britt and *Helietta apiculata* [42,43]; Gambogic acid (**22**), previously isolated from *Garcinia hanburyi* plant [44]; the unsaturated fatty acid Punicic acid (**25**), previously isolated from pomegranate seed oil [47]; and finally, the triterpenoid saponin Soyasapogenol B 3-*O*-d-glucuronide (**27**), previously isolated from aerial parts of *Lathylus palustris* L. [49,50].

**Molecular docking study of *A. alopecuroides* metabolites for inhibition of α-amylase and α-glucosidase enzymes**

**Figure 4:**
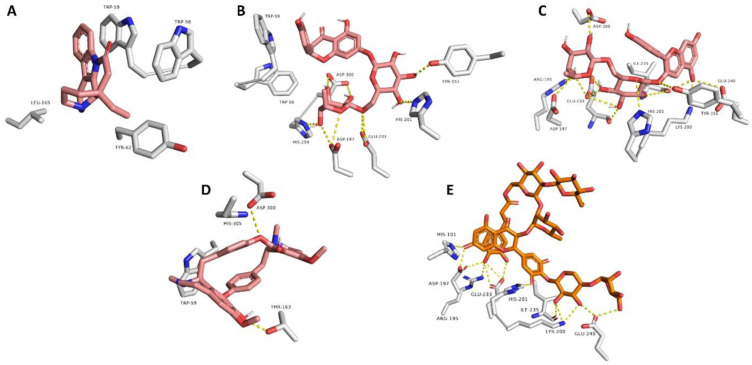
Docking poses of compounds with docking scores <−8.0 kcal/mol (i.e., Eburnamonine, Narirutin, Pelargonidin 3-*O*-rutinoside, and Isochondrodendrine along with the co-crystalized inhibitor Montbretin A) inside the human α-amylase (**A**–**E**, respectively).

In order to highlight the probably bioactive metabolites in AAE, all the dereplicated compounds were subjected to molecular docking study against both human α-amylase and α-glucosidase enzymes. All the dereplicated compounds displayed binding energies within the range of −4.5 to −9.1 Kcal/mol with the two enzymes (Table 2). The two alkaloids Eburnamonine (**2**) and Isochondrodendrine (**24**) as well as the two flavonoids Narirutin (**9**) and Pelargonidin 3-*O*-rutinoside (**10**) achieved the best binding scores with α-amylase enzyme. These compounds showed various binding modes inside the enzyme active site. Narirutin and Pelargonidin 3-*O*-rutinoside binding poses were comparable with that of the co-crystalized inhibitor Montbretin A (Figure 4B,C,E), where they established multiple H-bonds with TYR-151, ASP-197, HIS-201, GLU-233, HiS-299, and ASP-300. In addition, Narirutin established further hydrophobic interactions with TRP-58 and TRP-59. Narirutin was previously reported to have a potent role in diabetes management and control of its complications. This effect was confirmed via in vitro and docking studies against eight target proteins including α-amylase and α-glucosidase. In this report, Narirutin displayed hydrogen bonding interactions with both enzymes [13,15,27]. Moreover, variable flavonoids were previously tested for α-amylase and α-glucosidase inhibitory activity using in vitro testing and molecular docking approaches. The ligand–enzyme complexes for these compounds were studied, and it was concluded that the interactions occur mainly through H-bonding [54,55]. 

On the other side, the alkaloid Eburnamonine (**2**) established four hydrophobic interactions only inside the enzyme’s active site with TRP-58 TRP-59, TYR-62, and LEU-165 without any H-bonds (Figure 4A). Eburnamonine is an alkaloid that was previously isolated from several Vinica species and stated to contribute to the recorded antidiabetic effect of the total extract, via increasing hepatic utilization of glucose, suppressing the gluconeogenic enzymes, and regulation of insulin secretion, glucose, and lipid metabolism [17,56,57]. One more alkaloid, the isoquinoline alkaloid Isochondrodendrine (**24**), showed a remarkable result where it achieved the highest docking score (−9.1 kcal/mol) among all tested compounds. It established two H-bonds with THR-163 and ASP-300 together with a single hydrophobic interaction with TRP-59 (Figure 4D). Notably, this is the first report for the α-amylase enzyme inhibitory potential of Isochondrodendrine (**24**). However, other alkaloids, e.g., Topetecan and Cathine, were previously studied, and docking results concluded potent inhibitory activity against α-amylase enzyme [58].

**Figure 5:**
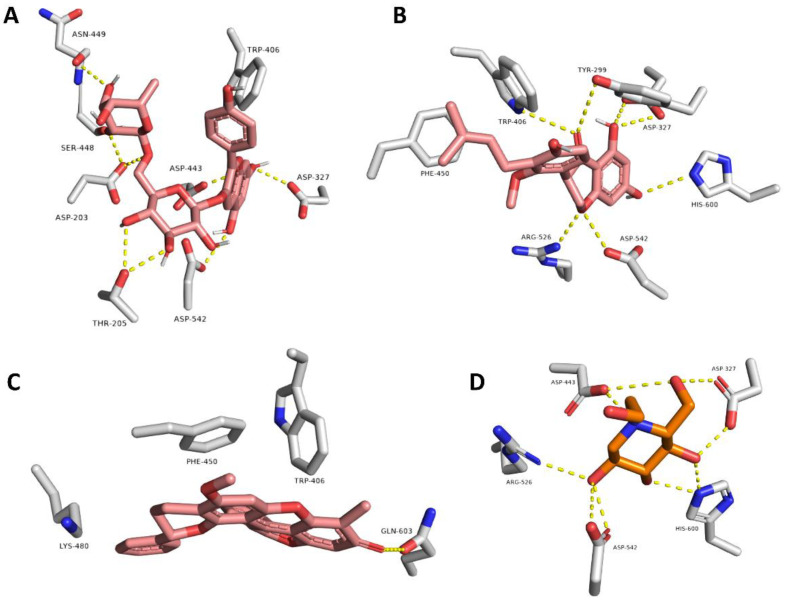
Docking poses of compounds with docking scores <−8.0 kcal/mol (i.e., Pelargonidin 3-*O*-rutinoside, Sophora isoflavanone A, and Dracorubin along with the co-crystalized inhibitor miglitol) inside the human α-glucosidase (**A**–**D**, respectively).

Regarding the α-glucosidase enzyme, the three flavonoids Pelargonidin 3-O-rutinoside (**10**), Sophora isoflavanone A (**14**), and Dracorubin (**23**) achieved the best scores for binding affinity. They showed different binding interactions inside the enzyme’s active site (Figure 5). Pelargonidin 3-*O*-rutinoside and Sophora isoflavanone A established interactions highly similar to that of the co-crystalized inhibitor Miglitol, where H-bonds were the predominant, e.g., with ASP-203, ASP-327, TRP-406, ASP-443, ASN-449, ARG-526, ASP-542, and HIS-600 (Figure 5A,B). On the other hand, Dracorubin’s major interactions were hydrophobic (e.g., with TRP-406, PHE-450, and LYS-480) in addition to a single H-bond with GLN-603 (Figure 5C). It is worth mentioning that this is the first report on the anti-enzyme activity of both compounds Sophora isoflavanone A and Dracorubin. On the other side, the anthocyanin compound, Pelargonidin 3-*O*-rutinoside, was previously reported to be a potent novel α-glucosidase inhibitor that can improve postprandial hyperglycemia [28,59]. Herein, Pelargonidin 3-*O*-rutinoside showed a promising dual inhibitory activity against both enzymes α-amylase and α-glucosidase, expressed as binding energy (−8.5 and −8.4 kcal/mol, respectively), which was better than that of both co-crystalized inhibitors of the two corresponding enzymes (−8.1 and −8.0 kcal, mol, respectively). The current results augmented the previous finding for α-glucosidase inhibitory activity in addition to providing further proof of the α-amylase inhibitory effect. Accordingly, this study nominated Pelargonidin 3-*O*-rutinoside as a potential antidiabetic agent.

**Bridging the metabolomic profiling of *A. alopecuroides* with its biological activity.**

In a research program dedicated to investigation of the biological potential and the phytochemical content of halophytes, *A. alopecuroides* was acknowledged for its characteristic antidiabetic activity [7]. Hence, it was crucial to explore the phytoconstituents in this species that might be responsible for such characteristic activity. In order to achieve this goal, the LC-HRMS/MS technique was employed. The current results addressed the richness of AAE with a wide variety of secondary metabolite classes. Among them, alkaloids and flavonoids are the most characteristic. Afterwards, the molecular docking technique was used to assess the antidiabetic potential of all dereplicated compounds. Docking results indicated the probable potential of most of the annonated compounds (binding energies ranging from −5 to −9); however, the most characteristic results were recorded by alkaloid and flavonoid constituents. Some of these constituents, e.g., Eburnamonine, Narirutin, and Pelargonidin 3-*O*-rutinoside, were previously reported for such activity, thus giving an interpretation for the observed antidiabetic activity of the crude extract. Other metabolites, such as Isochondrodendrine (−9.1 Kcal/mol, α-amylase), Sophora isoflavanone A (−9.1 Kcal/mol, α-glucosidase), and Dracorubin (−8.3 Kcal/mol, α-amylase), were noted for the first time as potential antidiabetic compounds. This finding adds more explanation for the observed activity of the total extract. Among the remaining dereplicated compounds, Pinoresinol glucoside was previously stated as a potent antidiabetic natural entity [32]. Herein, Pinoresinol glucoside displayed good inhibitory activity against α-amylase enzyme (−7.9 Kcal/mol) and α-glucosidase (−6.0 Kcal/mol). Other compounds such as Heliettin, 1,2-Dehydroreticuline, Epinorlycoramine, N-Feruloyltyramine, and Veraguensin also displayed good activity, expressed as binding energy in the range of −7.5 to −7.9 Kcal/mol. In conclusion, AAE contains a powerful mixture of phytoconstituents that could be considered, either individually or collectively, as a probable antidiabetic agents.

## Figures and Tables

**Table 1:** The LC-HRMS/MS dereplication results of *A. alopecuroides* methanolic extract.

No.	Tentative Identification	Rt	*m*/*z*	Formula	Ms/Ms-Fragmnets	Biological Source	Nature of Compounds	References
1	Epinorlycoramine	1.2	275.152	C_16_H_21_NO_3_	112.0874-124.0875-154.0978-190.098-202.0982-220.1087	*Narcissus leonensis* plant	Alkaloid	[16]
2	Eburnamonine	1.6	294.173	C_19_H_22_N_2_O	114.1028-145.0519	Leaves of *Kopsia zarutensi*	Alkaloid	[17]
3	3-Acetylnerbowdine	2.0	361.153	C_19_H_23_NO_6_	151.0774	Bulbs of *Nerine bowdenii*	Alkaloid	[18,19]
4	Arborinine	3.3	285.290	C_16_H_15_NO_4_	110.0375-135.0299-153.0546	Leaves of *Glycosmis parva*	Alkaloid	[20]
5	1,2-Dehydroreticuline	4.1	328.155	C_19_H_22_NO_4_	120.0817-132.0821-166.0880-178.0875	Bark and the root of *Xylopia parviflora*	Alkaloid	[21,22]
6	Versiconol acetate	6.7	402.095	C_20_H_18_O_9_	143.0711	Culture of *Aspergillus parasiticus*	Anthraquinone	[23]
7	Karwinaphthol B	7.0	288.136	C_17_H_20_O_4_	112.0879-117.0333-138.0664-145.0289	Roots of *Karwinskia humboldtiana*	Benzisochromans	[24]
8	Veraguensin	7.4	372.194	C_22_H_28_O_5_	177.0550-196.1444-222.1249	Leaves and root bark of *Nectandra turbacensis* (Kunth) Nees	Lignan	[25,26]
9	Narirutin	8.6	580.179	C_27_H_32_O_14_	313.0713-415.1029-433.1137	Citrus fruits	Flavanone glycoside	[27]
10	Pelargonidin 3-*O*-rutinoside	8.6	579.171	C_27_H_31_O_14_	313.0713-397.0918 415.1029-433.1137	Strawberries	Anthocyanin	[28]
11	Boschnaloside	8.8	344.147	C_16_H_24_O_8_	151.0742-177.0546-186.0527	*Boschniakia rossica* plant	Iridoid glycoside	[29,30]
12	Biochanin A-β-d-glucoside	9.0	446.121	C_22_H_22_O_10_	145.0288-175.0619-177.0551	*Trifolium pratense* L. plant	Isoflavone glycoside	[31]
13	Pinoresinol glucoside	9.1	520.194	C_26_H_32_O_11_	177.0552-184.0717-186.0523-191.0710	Prunes of *Prunus domestica* L.	Lignan	[32]
14	Sophora isoflavanone A	9.2	370.142	C_21_H_22_O_6_	145.0285-177.0553-284.0695	*Sophora tomentosa* L. plant	Isoflavone	[33]
15	Geniposide	9.2	388.137	C_17_H_24_O_10_	149.0614-151.0390-177.0552-186.0541	Fruit of *Gardenia* *jasminoides* Ellis	Iridoid glycoside	[34]
16	N-Feruloyltyramine	9.3	313.131	C_18_H_19_NO_4_	117.0339-145.0293-149.0607-162.0539	Fruits of *Lycium* *barbarum* (goji berries) *Bassia indica* and *A. alopecuroides* plants	Alkaloid	[10,35]
17	4-Hydroxycinnamyl alcohol 4-d-glucoside	9.3	312.121	C_15_H_20_O_7_	117.0339-145.0293-149.0607-	*Linum usitatissimum*, Linn. plant	Lignan	[36,37]
18	Coniferin	9.4	342.131	C_16_H_22_O_8_	137.0600-175.0763-177.0546-218.0794	*Paulownia tomentosa* bark	Lignan	[38,39]
19	Powelline	9.7	301.131	C_17_H_19_NO_4_	121.0652-135.0448-163.0395-180.0660	Leaves from *Crinum latifolium* L.	Alkaloid	[40]
20	Toxyl angelate	9.7	300.136	C_18_H_20_O_4_	121.0652-135.0448-145.0286-163.0395-	*Isocoma wrightii* plant	Banzofuran	[41]
21	Heliettin	10.1	314.152	C_19_H_22_O_4_	121.0657-145.0289-177.0553	Stem bark of *Helietta longifoliata* Britt	Furanochomarine	[42,43]
22	Gambogic acid	10.2	628.304	C_38_H_44_O_8_	121.0655-177.0549-201.0549-297.1123	*Garcinia hanburyi* plant	Phenolic acid (Xanthoid derivative)	[44]
23	Dracorubin	10.9	488.162	C_32_H_24_O_5_	121.0654-177.0565-201.0545-323.0921	Resin extracted from the tree *Dracaena draco*	Proanthocyanidine	[45]
24	Isochondrodendrine	12.1	594.273	C_36_H_38_N_2_O_6_	565.2679	*Cissampelos mucronate* and *Cissampelos pareira* plants	Alkaloids	[46]
25	Punicic acid	13.0	278.225	C_18_H_30_O_2_	107.0864-121.1017-133.1021-135.1160-149.1334	Pomegranate Seed Oil	Unsaturated fatty acid	[47]
26	Conessine	13.3	356.319	C_24_H_40_N_2_	121.1015-123.1154-135.1162-149.1330	*Holarrhena floribunda* G. Don. plant	Alkaloid	[48]
27	Soyasapogenol B 3-*O*-d-glucuronide	13.5	634.408	C_36_H_58_O_9_	133.0863-177.1134-247.2054-291.2317	Aerial parts of *Lathylus palustris* L.	Triterpenoid saponin	[49,50]

**Table 2:** Docking sores and estimated absolute binding free energies (in kcal/mol) of the dereplicated structures, along with those of the reported co-crystalized inhibitors.

No.	Tentative Identification	Binding Energy (kcal/mol)	
		α-Amylase	α-Glucosidase
1	Epinorlycoramine	−7.6	−6.2
2	Eburnamonine	−8.7	−6.4
3	3-Acetylnerbowdine	−7.2	−6.6
4	Arborinine	−7.5	−6.0
5	1,2-Dehydroreticuline	−7.4	−7.4
6	Versiconol acetate	−7.5	−7.1
7	Karwinaphthol B	−7.4	−5.9
8	Veraguensin	−7.5	−6.7
9	Narirutin	−8.5	−7.9
10	Pelargonidin 3-O-rutinoside	−8.5	−8.4
11	Boschnaloside	−7.1	−5.8
12	Biochanin A-β-D-glucoside	−7.2	−6.3
13	Pinoresinol glucoside	−7.9	−6.0
14	Sophora isoflavanone A	−7.4	−9.1
15	Geniposide	−7.2	−6.5
16	N-Feruloyltyramine	−7.3	−7.6
17	4-Hydroxycinnamyl alcohol 4-D-glucoside	−7.3	−6.8
18	Coniferin	−6.9	−5.7
19	Powelline	−6.7	−6.5
20	Toxyl angelate	−7.2	−6.4
21	Heliettin	−7.2	−7.1
22	Gambogic acid	−7.2	−7.1
23	Dracorubin	−7.8	−8.3
24	Isochondrodendrine	−9.1	−7.0
25	Punicic acid	−5.6	−5.7
26	Conessine	−4.8	−5.6
27	Soyasapogenol B 3-O-D-glucuronide	−4.5	−4.7
STD	Montbretin A	−8.1	….
	Miglitol	….	−8.0

## Data Availability

All Data are contained within the article and Supplementary Materials.

## Supplementary Materials

The following supporting information can be downloaded at: https://www.mdpi.com/article/10.3390/life12111852/s1. Detailed material and method of docking study. References [60,61,62,63] are cited in the supplementary materials. Figure S1: 2D diagram of the binding interactions of the twenty-seven compounds detected in *A. alopecuroides* against α-Glucosidase enzyme. Figure S2: 2D diagram of the binding interactions of the twenty-seven compounds detected in *A. alopecuroides* against α-Amylase enzyme.
