# Supplementary material for: Chemical Profiling and Molecular Docking Study of Agathophora alopecuroides
Source: Life (Basel). 2022 Nov 11;12(11):1852. doi: 10.3390/life12111852 (PMC9696702; doi:10.3390/life12111852)
Supplement: Supplementary file 1 [file life-12-01852-s001.zip › life-2008628-SI.pdf]

Chemical Profiling and Molecular Docking Study of Agathophora alopecuroides

Elham Amin [1,2], Mohamed Sadek Abdel-Bakky [3,4], Hamdoon A. Mohammed [1,5] and Marwa H. A. Hassan [2,*]
[1] Department of Medicinal Chemistry and Pharmacognosy, College of Pharmacy, Qassim University, Buraydah 51452, Saudi Arabia
[2] Department of Pharmacognosy, Faculty of Pharmacy, Beni-Suef University, Beni-Suef 62514, Egypt
[3] Department of Pharmacology and Toxicology, College of Pharmacy, Qassim University, Buraydah 51452, Saudi Arabia
[4] Department of Pharmacology and Toxicology, Faculty of Pharmacy, Al-Azhar University, Cairo 11751, Egypt
[5] Department of Pharmacognosy and Medicinal Plants, Faculty of Pharmacy, Al-Azhar University, Cairo 11751, Egypt
***** Correspondence: marwa.hassan@pharm.bsu.edu.eg or mh_elsefy@yahoo.com; Tel.: +2-012-7898-2288; Fax: +2-(082)-2317958

Materials and Methods:

Molecular Docking

1. Docking-based virtual screening

1.1. Ligand Structure Generation

OpenBabel v.3.1.1 [1] was used to convert the structures' SMILE codes to three-dimensional configurations that were subsequently subjected to a minimization of energy using the steepest descent technique with the same software. The minimization was performed by the force field MMFF94. Using AutoDockTools v.4.2, all torsions of the selected structures were assigned and their Gasteiger charges were provided for all studied atoms in structures [2].

1.2. Protein Structure Preparation

For docking screening, the human α-amylase and human α-glucosidase crystal structures (PDB codes: 4W93 and 3L4W, respectively) [3,4] were used. PDBfixer [5] was used to edit the downloaded structure, adding missing residues and atoms, and removing co-crystalized H2O and heteroatoms. Through AutoDock Tools v.4.2, polar hydrogen and Gasteiger charges were subsequently made available for both proteins.

1.3. Structural Docking

The docking process was carried out using the PyRx platform's built-in AutoDock Vina software [5,6]. According to the co-crystalized ligands of both enzymes, the docking search grid boxes were determined to perfectly enclose them with a 20 Å3 total size.

The grid box's coordinates were set to be x = -9.682; y = 4.274; z = -23.145 and x = 45.424; y = 92.375; z = 34.811, respectively. The level of exhaustion was held at 24. Pymol software was used to evaluate and display docking poses. Exhaustiveness was set to 24. Ten poses were generated for each docking experiment. Docking poses were analyzed and visualized using Pymol software [7]. The docking protocol was validated by re-docking the co-crystalized ligands (i.e. montbretin A and miglitol) into the active sites of both enzymes (i.e. α-amylase and α-glucosidase, respectively). The resulting top-scoring poses of both ligands were in good alignment with the co-crystalized ones with slight deviations (RMSDs = 1.27 and 1.04, respectively).

References:

1. O'Boyle, N.M.; Banck, M.; James, C.A.; Morley, C.; Vandermeersch, T.; Hutchison, G.R. Open Babel: An open chemical toolbox. Cheminform. J. **2011**, 3, 33.

2. Morris, G.M.; Huey, R.; Lindstrom,W.; Sanner, M.F.; Belew, R.K.; Goodsell, D.S.; Olson, A.J. AutoDock4 and AutoDockTools4: Automated docking with selective receptor flexibility. J. Comput. Chem. **2009**, 30, 2785.

3 Williams, L.K.; Zhang, X.; Caner, S.; Tysoe, C.; Nguyen, N.T.;Wicki, J.;Williams, D.E.; Coleman, J.; McNeill, J.H.; Yuen, V.; et al. The amylase inhibitor montbretin A reveals a new glycosidase inhibition motif. Nat. Chem. Biol. **2015**, 11, 691–696.

4 Sim, L.; Jayakanthan, K.; Mohan, S.; Nasi, R.; Johnston, B.D.; Pinto, B.M.; Rose, D.R. New Glucosidase Inhibitors from an Ayurvedic Herbal Treatment for Type 2 Diabetes: Structures and Inhibition of Human Intestinal Maltase-Glucoamylase with Compounds from Salacia reticulata. Biochemistry **2009**, 49, 443–451.

5. Eastman, P.; Friedrichs, M.S.; Chodera, J.D.; Radmer, R.J.; Bruns, C.M.; Ku, J.P.; Beauchamp, K.A.; Lane, T.J.; Wang, L.; Shukla, D.; et al. OpenMM 4: A Reusable, Extensible, Hardware Independent Library for High Performance Molecular Simulation. J. Chem. Theory Comput. **2013**, 9, 461. https://doi.org/ 10.1021/ct300857j.

6. Dallakyan, S.; Olson, A.J. Chemical Biology; Hempel, J.E.,Williams, C.H., Hong, C.C., Eds.; Springer: New York, NY, USA, 2015; pp. 243–250. https://doi.org/10.1007/978-1-4939-2269-7_19.

7. Seeliger, D.; de Groot, B.L. Ligand docking and binding site analysis with PyMOL and Autodock/Vina. J. Comput. Aided Mol. Des. **2010**, 24, 417–422. https://doi.org/10.1007/s10822-010-9352-6.

<u>Supplementary Figures</u>:

Figure S1: 2D diagram of the binding interactions for the identified metabolite (**1-27**) from *A. alopecuroides* with the active site residues of the α-glucosidase receptor.

Figure S2: 2D diagram of the binding interactions for the identified metabolite (**1-27**) from *A. alopecuroides* with the active site residues of the α-amylase receptor.

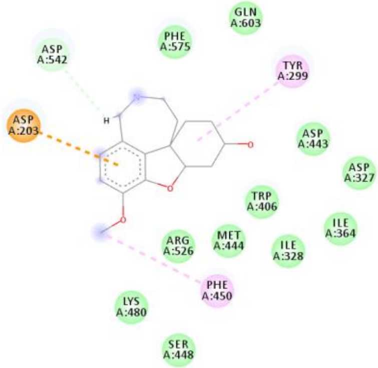

Epinorlycoramine (**1**)

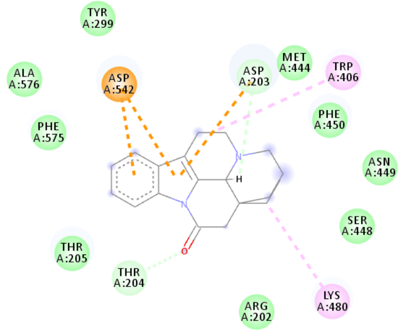

Eburnamonine (**2**)

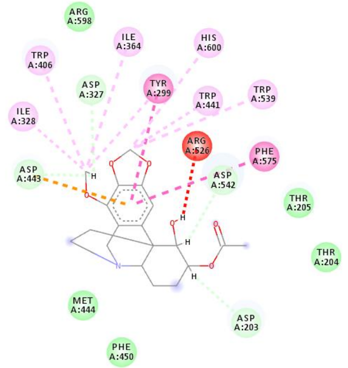

3-Acetylnerbowdine (**3**)

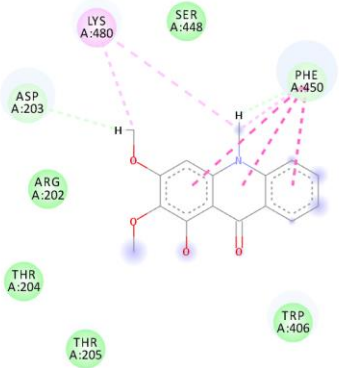

Arborinine (**4**)

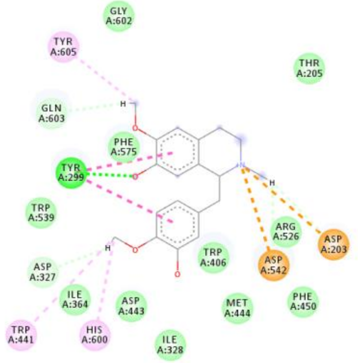

1,2-Dehydroreticuline (**5**)

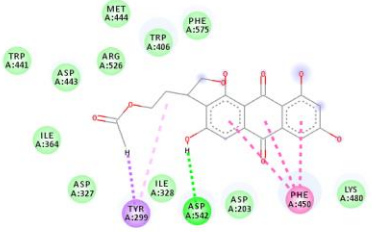

Versiconol acetate (**6**)

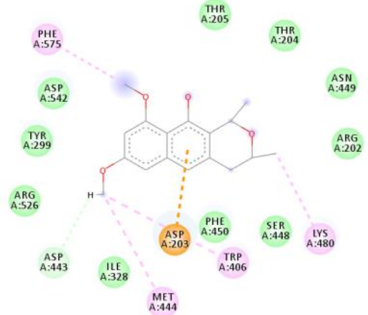

Karwinaphthol B (**7**)

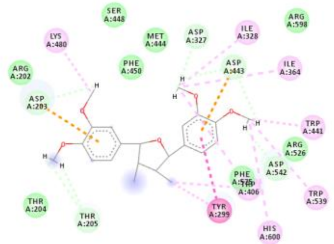

Veraguensin (**8**)

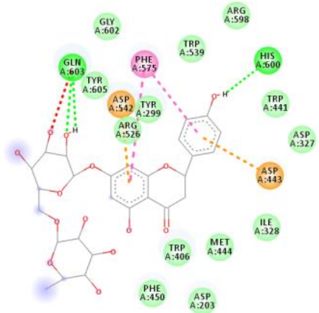

Narirutin (**9**)

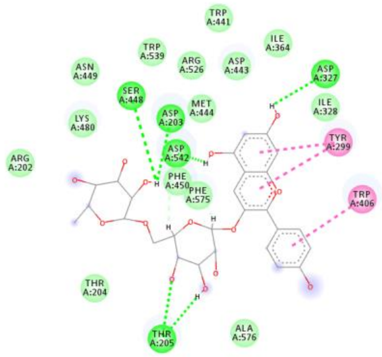

Pelargonidin 3-O-rutinoside (**10**)

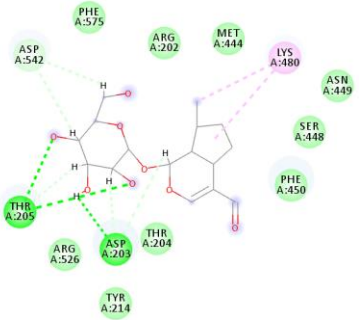

Boschnaloside (**11**)

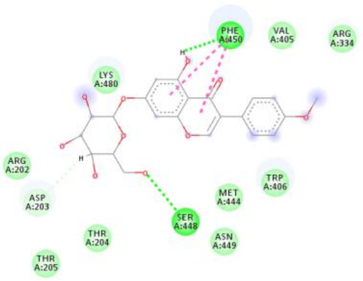

Biochanin A-B-D-glucoside (**12**)

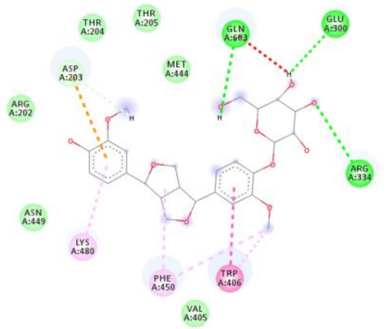

Pinoresinol glucoside (**13**)

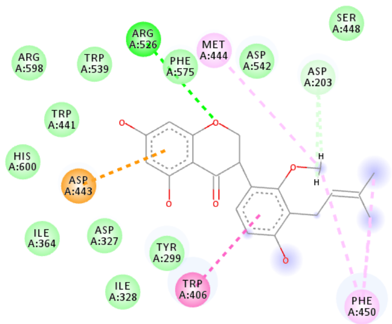

Sophora isoflavanone A (**14**)

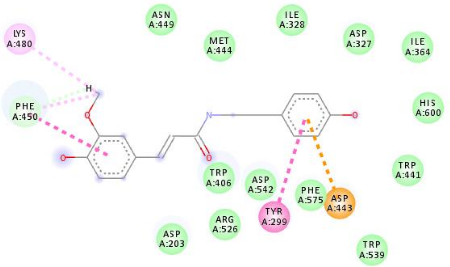

N-Feruloyltyramine (**15**)

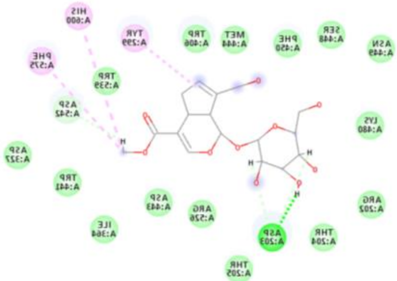

Geniposide (**16**)

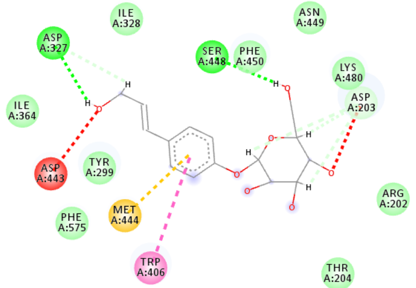

4-Hydroxycinnamyl alcohol 4-D-glucoside (**17**)

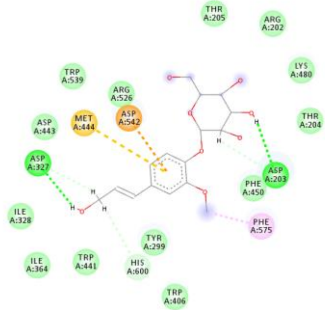

Coniferin (**18**)

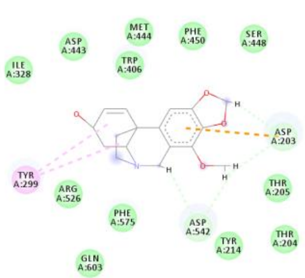

Powelline (**19**)

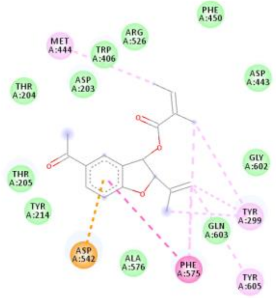

Toxyl angelate (**20**)

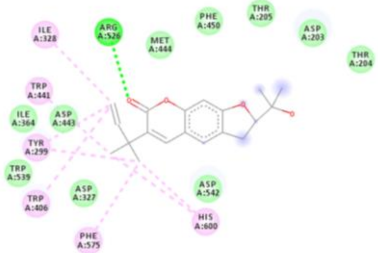

Heliettin (**21**)

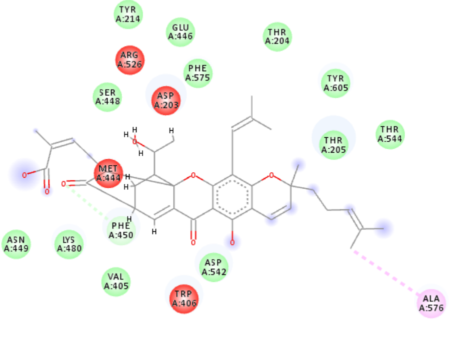

Gambogic acid (**22**)

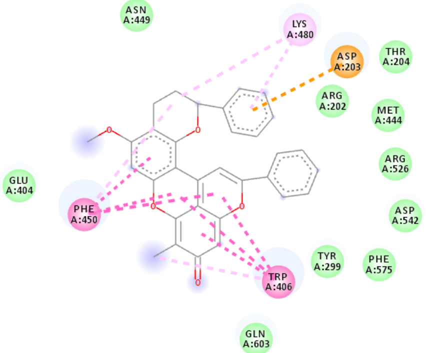

Dracorubin (**23**)

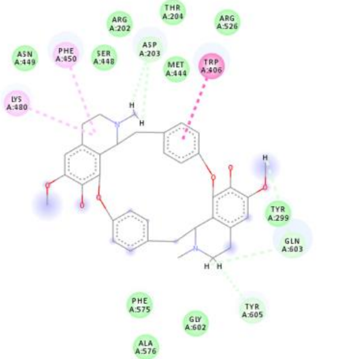

Isochondrodendrine (**24**)

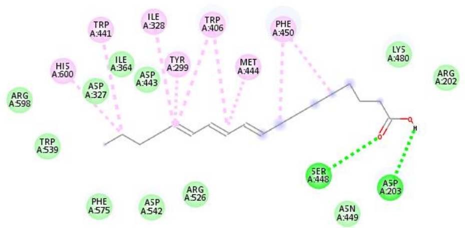

Punicic acid (**25**)

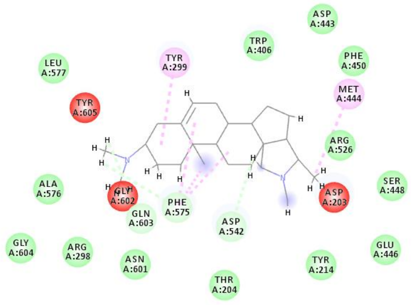

Conessine (**26**)

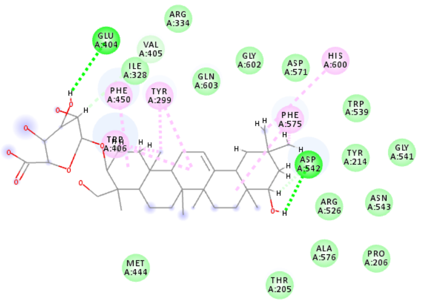

Soyasapogenol B 3-O-D-glucuronide (**27**)

Figure S1. 2D diagram of the binding interactions for the identified metabolite (**1-27**) from *A. alopecuroides* with the active site residues of the α-glucosidase receptor.

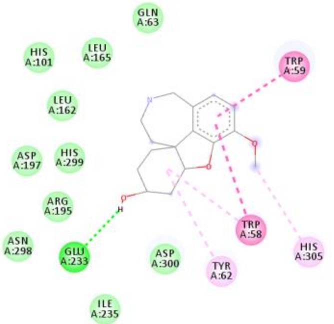

Epinorlycoramine (**1**)

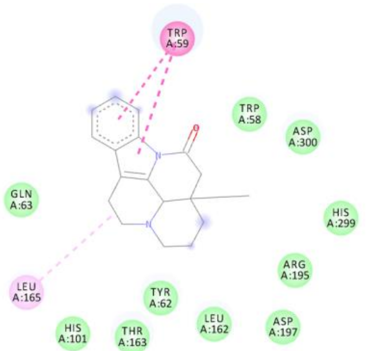

Eburnamonine (**2**)

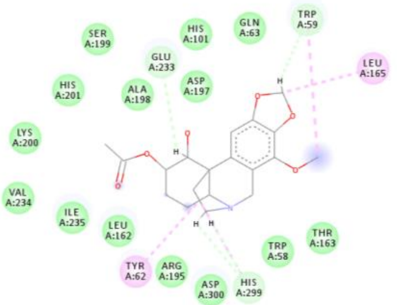

3-Acetylnerbowdine (**3**)

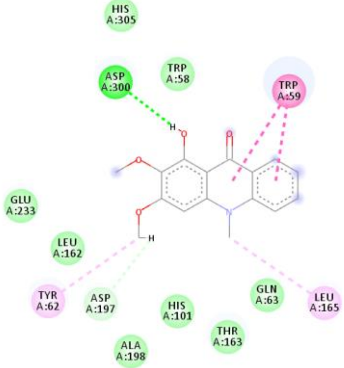

Arborinine (**4**)

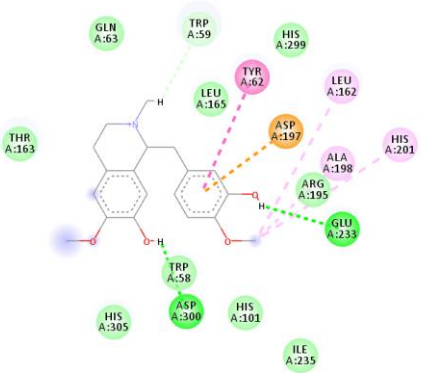

1,2-Dehydroreticuline (**5**)

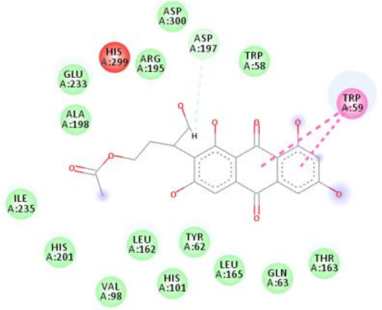

Versiconol acetate (**6**)

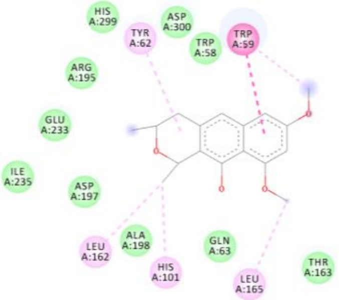

Karwinaphthol B (**7**)

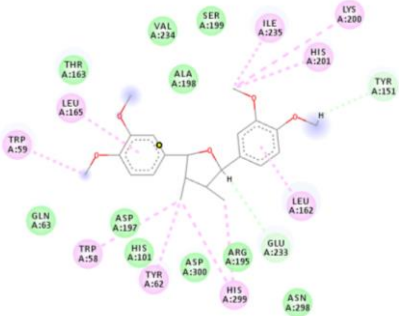

Veraguensin (**8**)

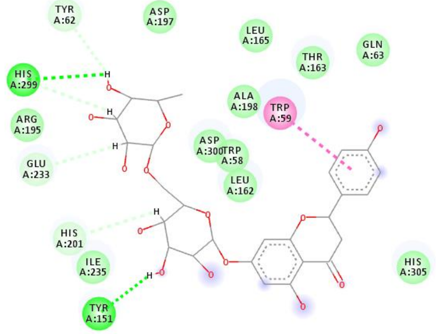

Narirutin (**9**)

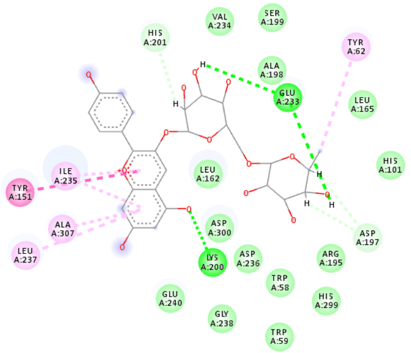

Pelargonidin 3-O-rutinoside (**10**)

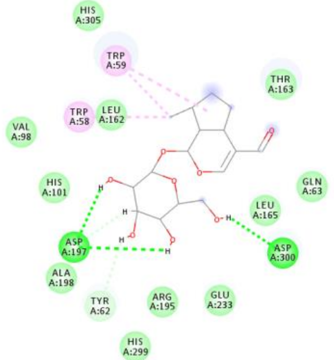

Boschnaloside (**11**)

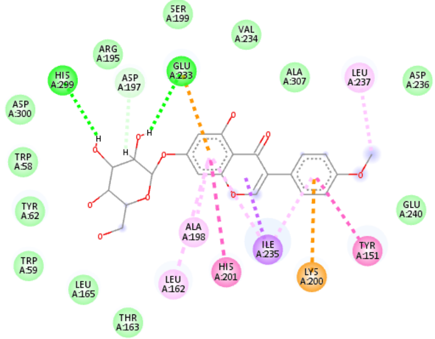

Biochanin A-B-D-glucoside (**12**)

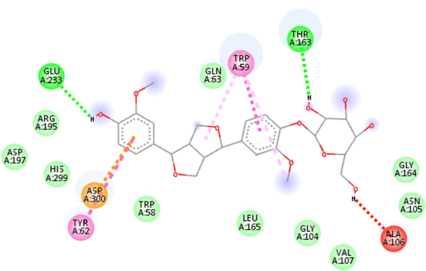

Pinoresinol glucoside (**13**)

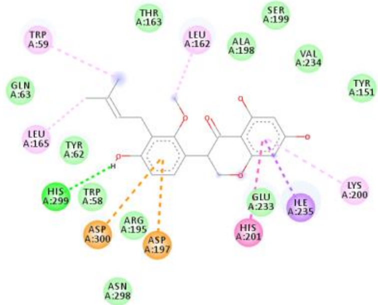

Sophora isoflavanone A (**14**)

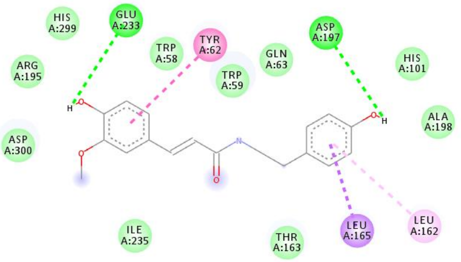

N-Feruloyltyramine (**15**)

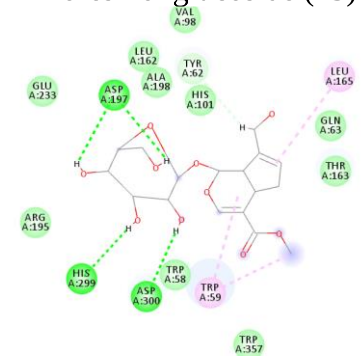

Geniposide (**16**)

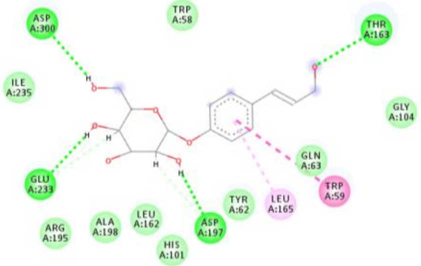

4-Hydroxycinnamyl alcohol 4-D-glucoside (**17**)

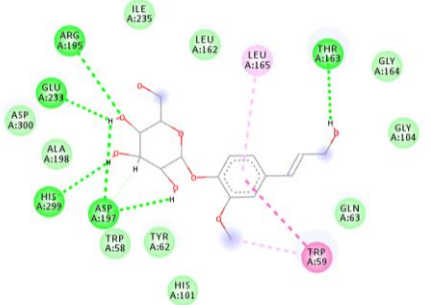

Coniferin (**18**)

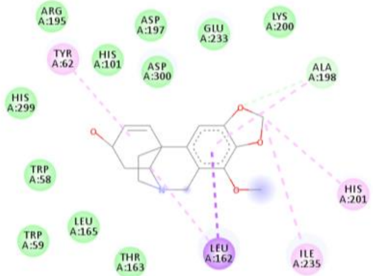

Powelline (**19**)

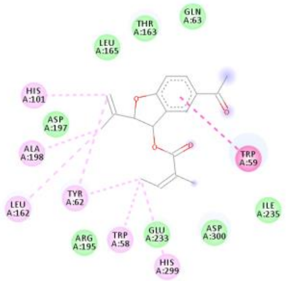

Toxyl angelate (**20**)

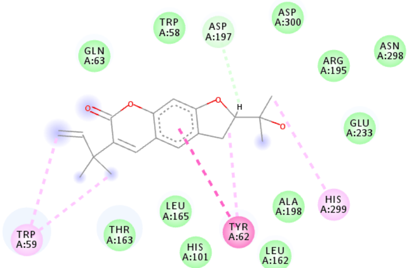

Heliettin (**21**)

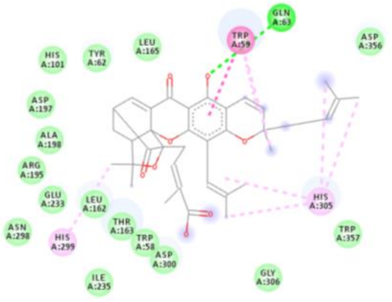

Gambogic acid (**22**)

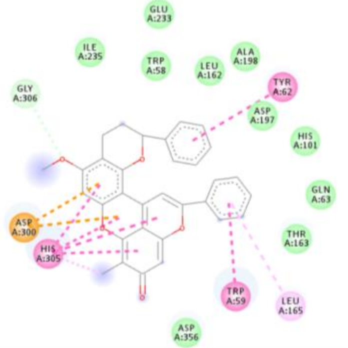

Dracorubin (**23**)

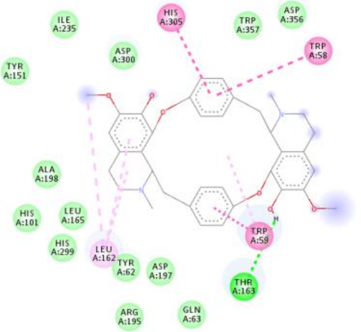

Isochondrodendrine (**24**)

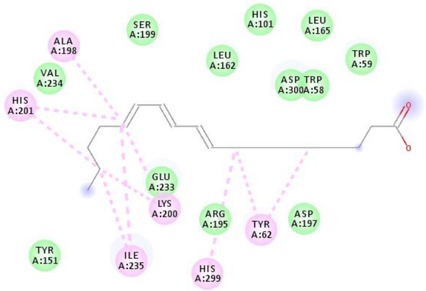

Punicic acid (**25**)

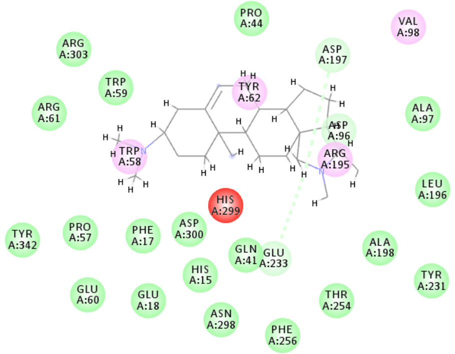

Conessine (**26**)

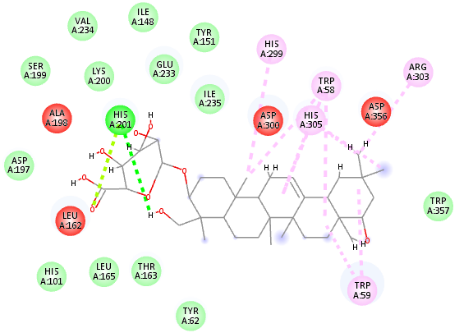

Soyasapogenol B 3-O-D-glucuronide (**27**)

Figure S2. 2D diagram of the binding interactions for the identified metabolite (**1-27**) from *A. alopecuroides* with the active site residues of the α-amylase receptor.
